# Novel Mutations in a Lethal Case of Lymphomatous Adult T Cell Lymphoma with Cryptic Myocardial Involvement

**DOI:** 10.3390/curroncol28010079

**Published:** 2021-02-06

**Authors:** Taraneh Hashemi Zonouz, Rami Abdulbaki, Bidhan C. Bandyopadhyay, Victor E. Nava

**Affiliations:** 1 Pathology and Laboratory Service, Veterans Affairs Medical Center, 50 Irving Street, NW, Washington, DC 20422, USA; thz62@email.gwu.edu (T.H.Z.); Rabdulbaki@gwu.edu (R.A.); 2 Department of Pathology, George Washington University, Washington, DC 20037, USA; 3 Calcium Signaling Laboratory, Veterans Affairs Medical Center, 50 Irving Street, NW, Washington, DC 20422, USA; bidhan.bandyopadhyay@va.gov; 4 Division of Renal Diseases & Hypertension, Department of Medicine, The George Washington University, Washington, DC 20037, USA

**Keywords:** adult T-cell leukemia/lymphoma, diabetic cardiomyopathy, next-generation sequencing, point mutations, TCR/NF-kB

## Abstract

The autopsy of a 65-year-old diabetic African American male revealed significant left myocardial involvement by adult T-cell leukemia/lymphoma (ATLL) despite normal pre-mortem fluorodeoxyglucose (FDG) uptake by positron emission tomography/computed tomography (PET/CT). Due to pre-existing diabetic cardiomyopathy with reduced ejection fraction (EF) and compatible imaging studies, cardiac lymphomatous involvement was not suspected. While peripheral blood was negative for leukemia, next-generation sequencing of a lymph node revealed at least eight novel mutations (AXIN1, R712Q, BARD1 R749K, CTNNB1 I315V, CUX1 P102T, DNMT3A S199R, FGFR2 S431L, LRP1B Y2560C and STAG2 I771M). These findings underscore a diagnostic pitfall in a rare lymphomatous variant of ATLL infiltrating myocardium and contribute to its molecular characterization.

## 1. Introduction

The World Health Organization (WHO) defines adult T-cell leukemia/lymphoma (ATLL) as a mature T-cell neoplasm composed of highly pleomorphic lymphoid cells and thought to be initiated by the retrovirus human T-cell leukemia virus type-1 (HTLV-1), which is necessary but not sufficient for its pathogenesis [1,2]. In North America, out of the four clinical variants of the disease (acute, lymphomatous, chronic and smoldering), the first two are the most common (~90% of cases) and carry a worse prognosis [3]. The lymphomatous type is characterized by prominent lymphadenopathy without peripheral blood involvement and by less frequent dermatologic manifestations or hypercalcemia [3]. Heart involvement by ATLL tends to be asymptomatic or oligosymptomatic. In autopsy series, few patients show cardiac symptoms like shortness of breath or palpitations, and massive cardiomegaly with extensive myocardial lymphomatous infiltration has seldom been reported [4,5,6]. 

The epidemiology of HTLV-1 in ATLL has been extensively studied. Geographic ATLL clustering overlaps endemic areas for the virus, such as southwestern Japan, the Caribbean basin and parts of central Africa. HTLV-1 is transmitted through blood products and breast feeding, and development of the disease follows a long (over 30 years) latency period after infection [7], which suggest that besides the causal role of viral oncogenes, host genetic and epigenetic factors are also necessary for tumorigenesis [8]. Two HTLV-1 proteins Tax and basic leucine zipper (HBZ) lead to transcriptional activation and T-cell proliferation, and both are able to transform in mouse models [9,10]. Tax-induced T-cell receptor (TCR)/nuclear factor kB (NF-kB) signaling has been well-documented in the development of ATLL [11]. However, Tax expression is silenced in a high proportion of ATLL cases, which illustrates its dispensable role in lymphomagenesis. In contrast, HBZ is the only viral protein consistently expressed in ATLL, and plays pleotropic roles modulating cell growth, T-cell differentiation and immune response, which contribute to oncogenesis. While most studies on the genetic alterations in ATLL have focused on Asian cases, recently, North American cases were analyzed in detail, demonstrating a unique frequency of epigenetic and histone modifying gene changes [12]. Conversely, the frequency of mutations in the JAK/STAT and the TCR/NF-kB pathway genes were lower when compared with the Japanese cases [12]. Therefore, it would be important to further analyze North American cases to confirm these findings, and to identify preclinical druggable target candidates. Here we present a lethal lymphomatous case of North American ATLL with extensive cardiac involvement and next-generation sequencing (NGS) data demonstrating several novel mutations that may hold promise in the diagnostic and therapeutic strategy. 

## 2. Result

A 65-year-old diabetic African American male followed at the Washington DC Veterans Affairs Medical Center for hypertension and complications of diabetes mellitus, including heart failure with 40% ejection fraction (EF), chronic renal failure and neuropathy, developed poor appetite, abdominal pain and weight loss of 15 pounds (6.8 kg) in May 2019. The patient was hospitalized due to exertional orthopnea, severe gastrointestinal reflux disease and worsening shortness of breath. 

Physical examination revealed mild obesity, unremarkable pulmonary and cardiovascular examination and no organomegaly or palpable lymphadenopathy. Imaging studies (including CT of the abdomen and pelvis with contrast) revealed infiltrating hypodense masses involving both kidneys, mesenteric lymphadenopathy and innumerable ground glass nodules with random distribution in the lung bases. The electrocardiogram was unremarkable. Multigated acquisition scan showed concentric left ventricular wall hypertrophy and hypokinesia, compatible with known cardiomyopathy. PET scan revealed innumerable foci of intense fluorodeoxyglucose (FDG) uptake in lungs, peritoneal cavity, kidneys, nodal basins (above and below the diaphragm), subcutaneous soft tissues and bones. However, normal FDG uptake within the myocardium was detected. Peripheral white blood cell counts were within the normal range. Serum calcium, sodium and potassium were also normal. Lactate dehydrogenase was markedly elevated at 1600 U/L, and serum HTLV-I antibody test was positive.

An enlarged supraclavicular lymph node was excised, and the diagnosis of ATLL was made based on histomorphology and positivity for CD45, CD2, CD3, CD4 and CD25 by immunohistochemistry. Ki-67 proliferative rate was very high (>80%). Other markers, including CD7 (aberrant loss), CD8, CD10, CD56, EBV and CD30, were negative. Careful examination of peripheral blood failed to detect atypical large lymphocytes, which was confirmed by a flow cytometric analysis showing no lymphomatous involvement. The diagnosis of lymphomatous variant of ATLL was confirmed, and NGS was performed by Foundation Medicine (comprehensive DNA and RNA analysis) and interpreted following the guidelines of the American College of Medical Genetics. This analysis revealed an intermediate tumor mutational burden (nine mutations per megabase) and stable microsatellite status. The following somatic mutations (Table 1) were also identified: *AXIN1* R712Q, *BARD1* R749K, *CBL* H42_L43insH, *CD36* amplification, *CDK6* amplification, *CTNNB1* I315V, *CUX1* (P102T and R44W), *DNMT3A* S199R, *FAS* D228fs*2, *FGFR2* S431L, *GATA3* loss exons 4–6, *HGF* amplification, *HIST1H2AM* loss, *HIST1H2BO* loss, *IRF4* amplification, *LRP1B* (D1063N and Y2560C), *NF1* S665F, *PCLO* amplification, *SDHD* F34C, *SMO* G24A, *STAG2* I771M and *TP53* H193L.

**Figure 1:**
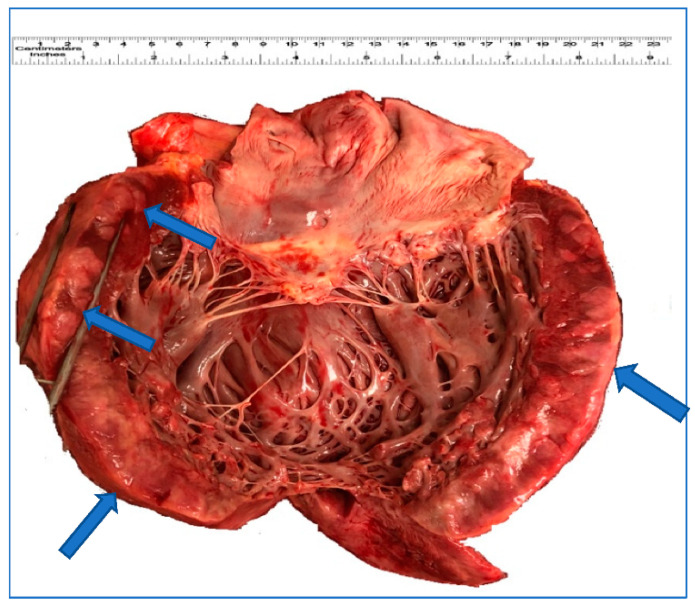
Gross appearance of the heart. The left myocardium shows and focal thickening and white-brown discoloration (arrow) that corresponds to leukemic infiltration.

**Figure 2:**
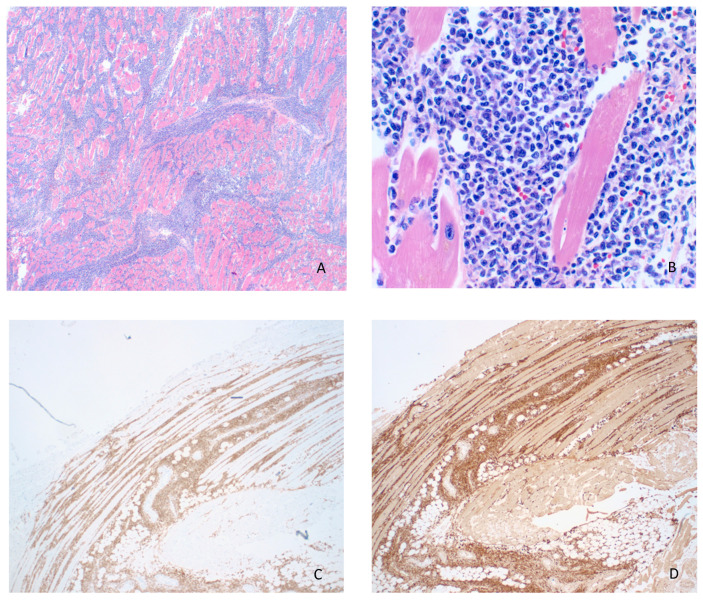
(**A**) Adult T-cell leukemia/lymphoma (ATLL) infiltration of endocardium and myocardium (H&E, 20×). (**B**) Higher magnification of infiltrating lymphoma showing pleomorphism, scant eosinophilic cytoplasm, irregular nuclear contours with coarse chromatin and variably prominent nucleoli (H&E, 200×). The lymphoma cells were strongly positive for CD4 (**C**) and CD25 (**D**) by immunohistochemistry (20×).

The patient underwent two cycles of chemotherapy (ESHAP-etoposide, methylprednisolone, cytarabine and cisplatin), but unfortunately had a complicated post-treatment course and developed tumor lysis syndrome, gastrointestinal bleeding, exacerbation of heart failure and unresolved acute kidney injury. Despite intensive care and cardiopulmonary resuscitation, the patient developed cardiac arrest and died 45 days after hospitalization. An autopsy revealed extensive lymphomatous involvement of multiple lymph nodes, including mesenteric, mediastinal, retroperitoneal, cervical and periaortic. In addition, significant involvement of the left myocardium (Figure 1) and multiple other organs (bilateral lungs, stomach, bowels, pancreas, bilateral kidneys, bilateral adrenals and prostate) and patchy involvement of diaphragm, skin and muscle was demonstrated by histomorphology and immunohistochemistry (including sections from the cardiac lymphoma shown in Figure 2).

## 3. Discussion

ATLL is a malignant lymphoproliferative neoplasm of mature T-cells initiated by monoclonal integration of HTLV-1 in the genome of lymphocytes, which leads to complex multistep events occurring during a long latency period. Despite a greater understanding of the pathogenesis of ATLL, curative treatment is lacking, and the overall prognosis remains dismal.

According to the Shimoyama classification, four variants of ATLL are recognized: acute, lymphomatous, chronic and smoldering [3]. The lymphomatous form is usually clinically aggressive and presents with advanced disease, including prominent lymphadenopathy and infrequent hypercalcemia, as seen in our patient. Although extensive involvement of the spleen and skin are common in lymphomatous ATLL, we did not observe these features. Instead, we detected patchy dermal involvement and extensive myocardial infiltration by ATLL, a rare event typically associated with ominous prognosis and concomitant lymphomatous involvement of the lung [6], as our necropsy illustrated. Of note, cardiac ATLL can be commonly missed pre-mortem even with modern imaging (as performed here) and is associated with chemoresistance [6]. Cutaneous involvement in our case was subclinical, as demonstrated by immunohistochemistry of a few tiny lymphoid aggregates found by microscopic examination, which is also unusual in lymphomatous ATLL.

Despite substantial progress in recent years, the molecular pathogenesis of ATLL remains unclear. After monoclonal integration of HTLV-1 and expression of viral oncogenes, such as Tax and HBZ, dysregulation of signaling pathways related to T-cell proliferation/differentiation and immune surveillance leads to leukemogenesis. Interestingly, Tax is dispensable for transformation and inactivated in a high proportion of cases [9]. However, HBZ is the only viral protein that remains consistently expressed in ATLL cases. Activation of PI3K, JAK/STAT and TCR/NF-kB pathways by genetic and epigenetic mechanisms seem to be crucial for tumor progression, and specific genes are commonly mutated, including CARD22, CCR4, CCR7, CDKN2A (p16), CDK2B (p15), EP300, FAS, FYN, GATA3, IRF4, PLCG1, PRKCB, TP53, STAT3 and VAV1. Accordingly, we observed mutations in genes of the TCR/NF-κB pathway (CBL, CUX1 and FAS), implicated in unrestricted and persistent NF-κB activation leading to the development of autoimmune diseases and neoplasms [13]. Such disbalance between T-cell activation and excessive NF-κB stimulation may have initiated deleterious consequences of TCR signaling, which, without negative regulation of nuclear signaling, may contribute to tumor progression. Interestingly, genetic alterations in genes associated with WNT/beta-catenin (AXIN1, CTNNB1 and SMO) and the RAS/MAPK (NF1), which are less commonly associated with ATLL, were also detected, suggesting possible unique pathogenic associations. As expected, several mutated genes that play a role in genomic stability (BARD1, TP53 and STAG2) and/or transcriptional regulation (HIST1H2AM, HIST1H2BO, IRF4 and GATA3) were seen. Also, genes directly involved in cell cycle control (CDK6) and soluble growth factors (HCF) had mutations in this tumor. Furthermore, trophic pathways related to surface receptors seem to be involved in our case, as suggested by mutations in CD36 (a lipid scavenger receptor) [14], LRP1B (a member of the low-density lipoprotein receptor family) [15] and FGFR2 (fibroblast growth factor receptor 2/CD332) [12]. Furthermore, additional epigenetic regulation may be represented by mutations in DNMT3A, a methyltransferase that may modulate gene expression by altering histones [16]. Finally, we found alterations in genes that have an unclear oncogenic mechanism of action, such as SDHD (a well-known driver gene in Cowden/Cowden-like syndromes) [17] and PCLO (a scaffold protein of the presynaptic cytomatrix at the active zone) [18].

Mutations in some of these genes (CBL, CDK6, DNMT3A, FAS, FGFR2, GATA3, HGF, IRF4, TP53 and STAG2) are well known in association with ATLL [12], but the specific alterations found are novel (CBL H42_L43insH, DNMT3A S199R, FGFR2 S431L, GATA3 loss of exons 4–6 and STAG2 I771M). Other genes have been described [18,19,20] in association with different hematopoietic or solid tumors (AXIN1, BARD1, CUX1, NF1, PCLO, SDHD and SMO), but are reported here for the first time in association with ATLL. Of interest, eight mutations (AXIN1, R712Q, BARD1 R749K, CTNNB1 I315V, CUX1 P102T, DNMT3A S199R, FGFR2 S431L, LRP1B Y2560C and STAG2 I771M) have not been reported before to the best of our knowledge after extensive literature/database searches. Current molecular pathogenesis of ATLL postulates important processes related to HTLV-1-mediated proliferation and immune evasion based on unclear stochastic genetic alterations necessary for full-blown disease. Accordingly, mutations belonging to major well-characterized pathways were found in this study. Focusing on the novel point mutations in the TCR/NF-kB (CUX1 P102T), epigenetic/methylation regulatory (DNMT3A S199R) and genetic stability (BARD1 and STAG2) pathways, our findings suggest commonalities with prior reports [12]. Similarly, various novel mutations belonging to the category of trophic proliferative signaling (FGFR2 S431L and LRP1B Y2560C) were sequenced. However, we also found novel mutations in the WNT/beta-catenin pathway (AXIN1, R712Q and CTNNB1 I315V), which is underappreciated in ATLL. Overexpression of Wnt5a (an activator of beta-catenin/CTNNB1 not found in our case) has been proposed as a mediator of hypercalcemia [21] and suggests that the beta-catenin (CTNNB1 I315V) defect we observed may have abrogated this mechanism, since our patient remained normocalcemic. Likewise, AXIN1 inactivation may have contributed to such normocalcemic phenotype, since this tumor suppressor regulates G-protein coupled signaling upstream of beta-catenin [20].

In summary, we present a clinically unusual lethal case of ATLL with several novel mutations (AXIN1, R712Q, BARD1 R749K, CTNNB1 I315V, CUX1 P102T, DNMT3A S199R, FGFR2 S431L, LRP1B Y2560C and STAG2 I771M) and unexpected myocardial involvement, which probably contributed to the fatal outcome.

## Figures and Tables

**Table 1:** Somatic mutations identified by next-generation sequencing (NGS).

Gene	Somatic Mutation	Allele Frequency (%)
*AXIN1*	R712Q	44.94
*BARD1*	R749K	24.75
*CBL*	H42_L43insH	17.61
*CTNNB1*	I315V	76.45
*CUX1*	R44W	36.15
*CUX1*	P102T	26.84
*DNMT3A*	S199R	39.04
*FAS*	D228fs*2	45.37
*FGFR2*	S431L	26.61
*LRP1B*	D1063N	68.28
*LRP1B*	Y2560C	46.64
*NF1*	S665F	31.97
*SDHD*	F34C	29.79
*SMO*	G24A	48.98
*STAG2*	I771M	40.98
*TP53*	H193L	70.02
